# Where does the money go to? Cost analysis of gynecological patients with a benign condition

**DOI:** 10.1371/journal.pone.0254124

**Published:** 2021-07-09

**Authors:** Kristiina Pynnä, Pirjo Räsänen, Risto P. Roine, Piia Vuorela, Harri Sintonen

**Affiliations:** 1 Obstetrics and Gynecology, University of Helsinki and Helsinki University Hospital, Helsinki, Finland; 2 Hospital District of Helsinki and Uusimaa, External Evaluation Unit, Helsinki, Finland; 3 Group Administration, University of Helsinki and Helsinki University Hospital, Helsinki, Finland; 4 Department of Health and Social Management, University of Eastern Finland, Kuopio, Finland; 5 Obstetrics and Gynecology, University of Helsinki and Helsinki University Hospital, Biomedicum Helsinki, Helsinki, Finland; 6 City of Vantaa, Department of Health and Social Welfare, Vantaa, Finland; 7 Department of Public Health, University of Helsinki, Helsinki, Finland; Duke University, UNITED STATES

## Abstract

**Objectives:**

The impact of benign gynecological conditions on life of women and on costs for the society is high. The purpose of this study is to gain knowledge and understanding of costs of the treatment of these disorders in order to be able to improve the clinical care processes, gain insight into feasible savings opportunities and to allocate funds wisely.

**Methods:**

The healthcare processes of 311 women attending university or community hospitals in the Helsinki and Uusimaa Hospital District between June 2012 and August 2013 due to a benign gynecological condition were followed up for two years and treatment costs analysed.

**Results:**

Total direct hospital costs averaged 689€ at six months and 2194€ at two years. The most expensive treatment was that of uterine fibroids in the short term and that of endometriosis and fibroids later on. Costs did not depend on hospital size. Surgical operations caused nearly half of hospital costs. Productivity loss caused biggest expenses outside of the hospital. LNG-IUD (levonorgestrel-releasing intrauterine device) accounted for the largest pharmaceutical costs for patients. Hospital treatment was associated with a reduced need for outpatient services during follow-up.

**Conclusions:**

A majority of direct hospital costs arise over time. This stresses the need for prolonged healthcare management. To control costs, the need for repetitive doctors’ appointments, monitoring tests, and ward treatments should be carefully evaluated. Procedures not needing an operation theatre (for example hysteroscopy for polypectomy), should be done ambulatorily.

## 1. Introduction

The impact of common benign gynecological conditions on the life of women [1] and on the costs for the society is high [2–6]. Half of women suffer from a benign gynecological condition during their fertile life [7]. Nearly 80% of them see a doctor annually and one third spends one or more days in bed every year [7]. Especially physical, sexual and mental well-being is impaired [1, 8, 9]. Costs of benign gynecological conditions are high and similar to those of chronic diseases such as diabetes, Crohn’s disease, Parkinson’s disease, and rheumatoid arthritis [10, 11]. Gynecological bleeding disorders, for example, account for annual costs of $13 billion [8] and endometriosis for $22 billion (0.7% of US annual health care expenditure) in the US [12, 13].

Limited healthcare resources mandate that more focus is set on optimizing healthcare processes and on the cost-effectiveness and opportunity costs of treatment. As the ageing of the population and the desire for improved quality-of-life are associated with high healthcare costs [14], it is important to understand how the total costs build up to be able to cut unnecessary costs and to allocate resources wisely.

This study provides new, original, short- and long-term data on the healthcare processes and costs of treatment of common benign gynecological conditions in a publicly funded healthcare setting. Our study also reports the indirect costs of treatment and the more seldom reported other out-of-hospital costs. Furthermore, the study is set to answer whether the distribution of costs differs depending on the treated condition or the hospital size and whether hospital treatment diminishes the need to seek care elsewhere and thus decreases costs and indirect societal costs. This knowledge enables better recognition of patient groups whose treatment protocols need further development.

## 2. Materials and methods

A prospective observational cost study conducted in the obstetrics and gynecology departments of the hospitals of the Helsinki and Uusimaa Hospital District: the Helsinki University Hospital (comprising of three hospitals in the Helsinki metropolitan area) and four smaller community hospitals. All women treated for a benign gynecological condition in these hospitals between June 1^st^, 2012 and August 31^st^, 2013 were invited to participate. Patients with uterine fibroids or some other benign uterine neoplasms, endometriosis, adenomyosis, uterine prolapse, endometrial or cervical polyps, gynecological bleeding disorder, dysmenorrhea or lower abdominal/pelvic pain or some other benign uterine finding were included in the study. The patients were grouped according to the ICD-10 classification codes after clinician’s routine assessment. If a specific condition/pathology was found behind the presenting symptoms (for example a uterine fibroid or a uterine polyp causing abnormal uterine bleeding), the patient was categorized according to this primary diagnosis. If no apparent reason for the condition was found, the patient was grouped according to this idiopathic symptom (for example idiopathic menorrhagia in the bleeding disorder group).

Patients were treated according to typical clinical practice in the Finnish publicly funded (taxation based) healthcare system. The clinical care process was tracked regarding doctor’s appointments, laboratory examinations, imaging, pathological examinations, outpatient clinic procedures, surgical operations, and ward/inpatient treatment in the different patient groups. The direct hospital costs were acquired from the clinical patient administration database and are reported at the current cost level of the follow-up time (from June 2012 –August 2013 (study intake) until June 2014 –August 2015 (last follow-up survey). These direct hospital costs were analysed both in short-term (six-month follow-up) and in long-term (two-year follow-up). The average exchange rate during the study period varied between 1EUR = 1.11–1.33USD.

To take out-of-hospital costs into account, the patients were asked to fill in a questionnaire concerning the use of services outside of the hospital before treatment (before/when attending the first outpatient clinic appointment, baseline), and at the six-month and the two-year follow-up. Patients reported nurses’ and doctors’ appointments at primary health care centres or private clinics, laboratory examinations, imaging, treatment for the same condition in other hospitals or clinics, drug purchases (including pain killers (for example NSAIDs), blood loss diminishing medicines (for example tranexamic acid), progesterone derivates (hormonal products) and separately LNG-IUD (levonorgestrel-releasing intrauterine device)) and absence from work during a three-month time period before each follow-up. Healthcare unit costs, productivity costs in Finland [15, 16] and pharmaceutical prices were incorporated into patient-reported use of services to obtain estimates of total costs to the patient, hospitals and society throughout the follow-up.

### 2.1. Statistical analyses

Paired samples t-test was used to test the statistical significance of the change in costs during follow-up. The statistical significance of the differences in the mean costs between two unrelated groups was tested by independent samples t-test. Two-sided p-values < 0.05 were considered statistically significant. The data were analysed using SPSS for Windows statistical software version 22.0 (SPSS, Inc., Chicago, IL, USA).

### 2.2. Ethical approval

The Ethics Committee of the Helsinki University Central Hospital approved the study protocol (registration number 538/E0/02). The study was conducted in accordance with the 1964 Helsinki declaration and its later amendments. Informed written consent was obtained from all participants.

## 3. Results

Of the 1173 women invited to participate, 543 (46.3%) completed the baseline questionnaire. Of these, 425 (78.3%) answered also at six months and 397 (73.1%) at two years. The inclusion criteria (appropriate reason of treatment and having answered all questionnaires at all follow-ups) were met by 389 patients at six months and 311 patients at two years. Of them, 178 (57.2%) were treated in the university hospital and 133 (42.8%) in community hospitals. Patients were treated because of bleeding disorders (n = 94), uterine fibroids (n = 79), polyps (n = 68), dysmenorrhea/pelvic pain (n = 24), endometriosis (n = 23), uterine prolapse (n = 7) or some other benign uterine disorder (n = 16). The mean duration of the long-term follow-up was 24 months 11 days (SD±12days). The mean age of the patients at baseline was 49.6 years (SD±12.8, range 19 to 98).

### 3.1. Operative versus conservative treatment

Of all patients, 167 (46.3%) underwent surgery in an operation theatre. Of university hospital patients 44.4% (n = 79) and of community hospital patients 48.9% (n = 65) were treated operatively (difference not statistically significant). Patients with uterine fibroids or a prolapse were most often treated operatively (70.9% and 71.4%, respectively) and patients with bleeding disorders, pelvic pain or some other benign gynecological condition least often (31.9%, 20.8%, and 6.3%, respectively). The only statistically significant difference in operative treatment between the hospitals was that polyp patients were more often treated in the operation theatre in the community hospitals compared to the university hospital (66.7% versus 36.8%) (S1 Fig).

### 3.2. Direct hospital costs

**Fig 1:**
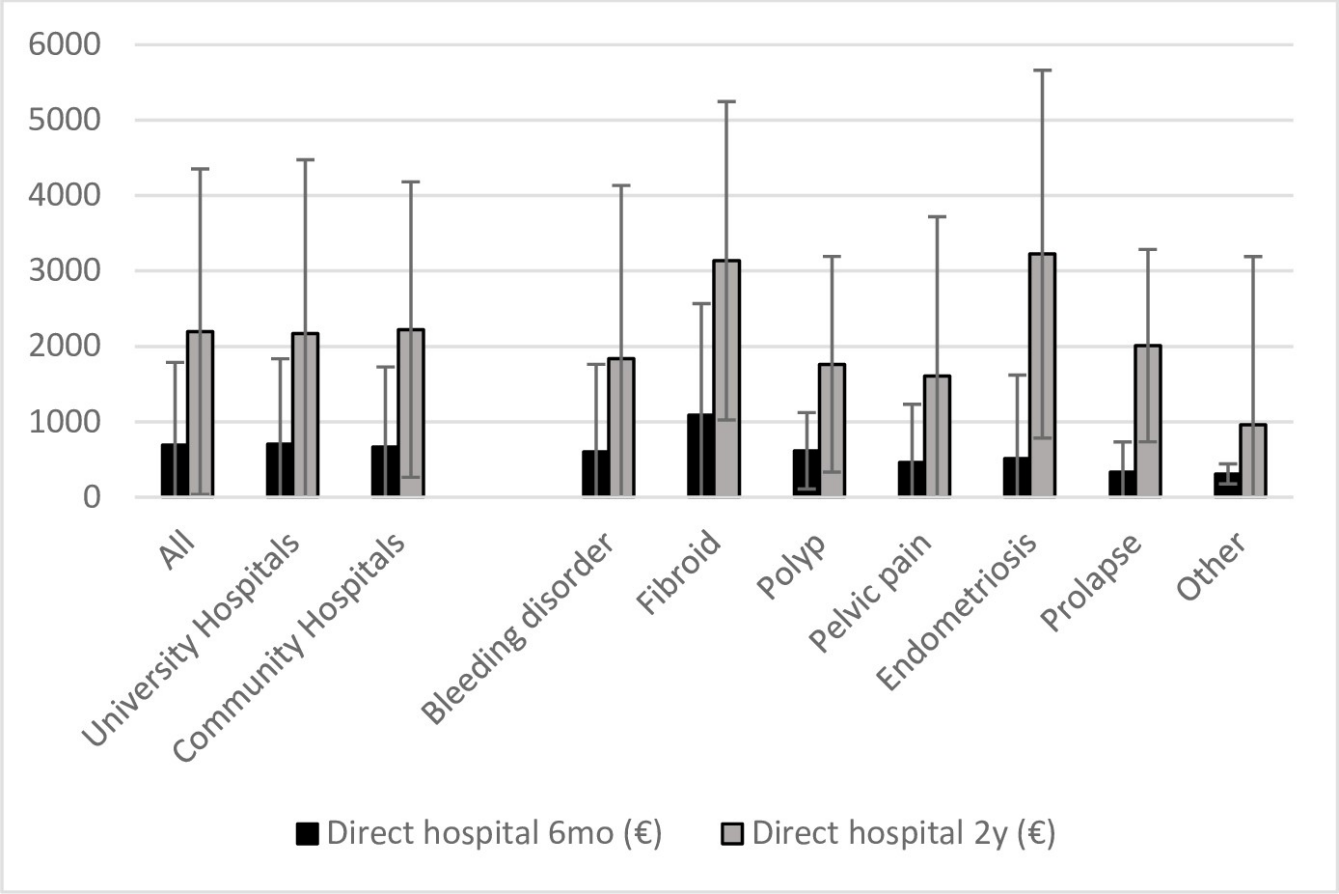
Direct mean hospital costs (€) according to hospital and diagnosis group (standard deviation represented by deviation lines).

The total direct costs averaged 688.6€ (SD±1098.8 €) at six months and 2193.8€ (SD±2158.6€) at two years. Treatment of uterine fibroids was the most expensive intervention at six months. At two years, the costs were highest in the endometriosis and uterine fibroids groups. The treatment of other benign gynecological conditions was less expensive during the whole follow-up (Fig 1). There was no statistically significant difference between the mean costs of treatment of the whole patient group in the university or community hospitals either at six months (704.7€ vs. 667.0€, respectively) or two years (2172.5€ vs. 2222.2€, respectively). More resources were used to treat endometriosis patients in the university hospitals compared to the community hospitals both at six months and two years (means 807.7€ vs. 190.0€ and 3958.4€ vs. 2421.3€, respectively). By contrast, the university hospitals spent less money on the treatment of polyps in by the two-year follow-up (means 1535.8€ vs. 2049.5€, respectively). However, these differences were not statistically significant (p = 0.19, p = 0.13 and p = 0.14, respectively) (Fig 1).

### 3.3. Breakdown of direct hospital costs

Nearly half of total direct costs (48.4%) was caused by surgery. Doctor’s appointments, inpatient/ward treatment, and outpatient procedures all accounted for similar proportions (13–17%) (Table 1). Doctor’s appointments generated more costs in community hospitals than in university hospitals. Otherwise, the cost distribution in the different hospitals was similar. A larger proportion of the costs was caused by ward and operation theatre treatment in patients with fibroids, endometriosis and prolapse when compared to the other diagnosis groups. Imaging caused relatively more costs in patients with pelvic pain or endometriosis. Outpatient procedures consumed relatively more resources in polyp patients and doctor’s appointments relatively more resources in the treatment of pelvic pain as compared to the other patient groups (Table 1).

**Table 1:** Direct hospital cost distribution according to hospital and diagnosis group.

	% of total hospital costs (statisticallysignificantdifference compared to other hospital type or other diagnosis groups)						
	Doctor’s appointment	Ward	Theatre	Outpatient clinic procedure	Imaging	Laboratory	Pathology
All	13,9%	12,6%	48,4%	16,9%	1,5%	2,2%	4,6%
University Hospitals	12,0%	13,1%	49,4%	16,7%	1,9%	2,4%	4,6%
Community Hospitals	16,5%	11,8%	47,1%	17,1%	1,1%	1,9%	4,5%
Bleeding disorder	16,0%	10,5%	44,5%	21,5%	0,9%	1,8%	4,8%
Fibroid	8,4%	16,6%	57,6%	9,4%	1,0%	2,0%	5,0%
Polyp	17,6%	4,9%	40,1%	29,4%	1,1%	1,5%	5,3%
Pelvic pain	28,5%	12,5%	30,1%	16,2%	5,7%	4,3%	2,6%
Endometriosis	12,6%	17,0%	52,3%	9,0%	3,5%	2,3%	3,3%
Prolapse	12,0%	14,3%	59,3%	11,4%	0,3%	1,4%	1,3%
Other	21,6%	6,9%	24,9%	31,8%	0,8%	10,7%	3,1%

### 3.4. Direct costs outside of hospitals and productivity costs

Mean direct costs outside of the hospitals (pharmaceuticals, healthcare services outside of the hospital) decreased in the whole study group significantly compared to baseline (450.2€ ±339.7) at both the six-month (309.0€ ±204.9) and two-year follow-ups (281.3€ ±421.9) (p<0.001). This decrease was seen in patients treated in university hospitals (482.5€ vs. 294.5€ and vs. 279.6€, p<0.001 and p<0.005), but not in those treated in community hospitals (407.1€ vs. 328.4€ and vs. 283.6€, p = 0.14 and p = 0.11). These direct costs were similar for patients treated conservatively or operatively. However, the productivity costs were temporarily, at six months, higher for patients treated operatively than for those treated conservatively (627.7€ vs 165.0€, p<0.005), but returned to low levels by two years (58.3€ vs. 117.7€).

**Fig 2:**
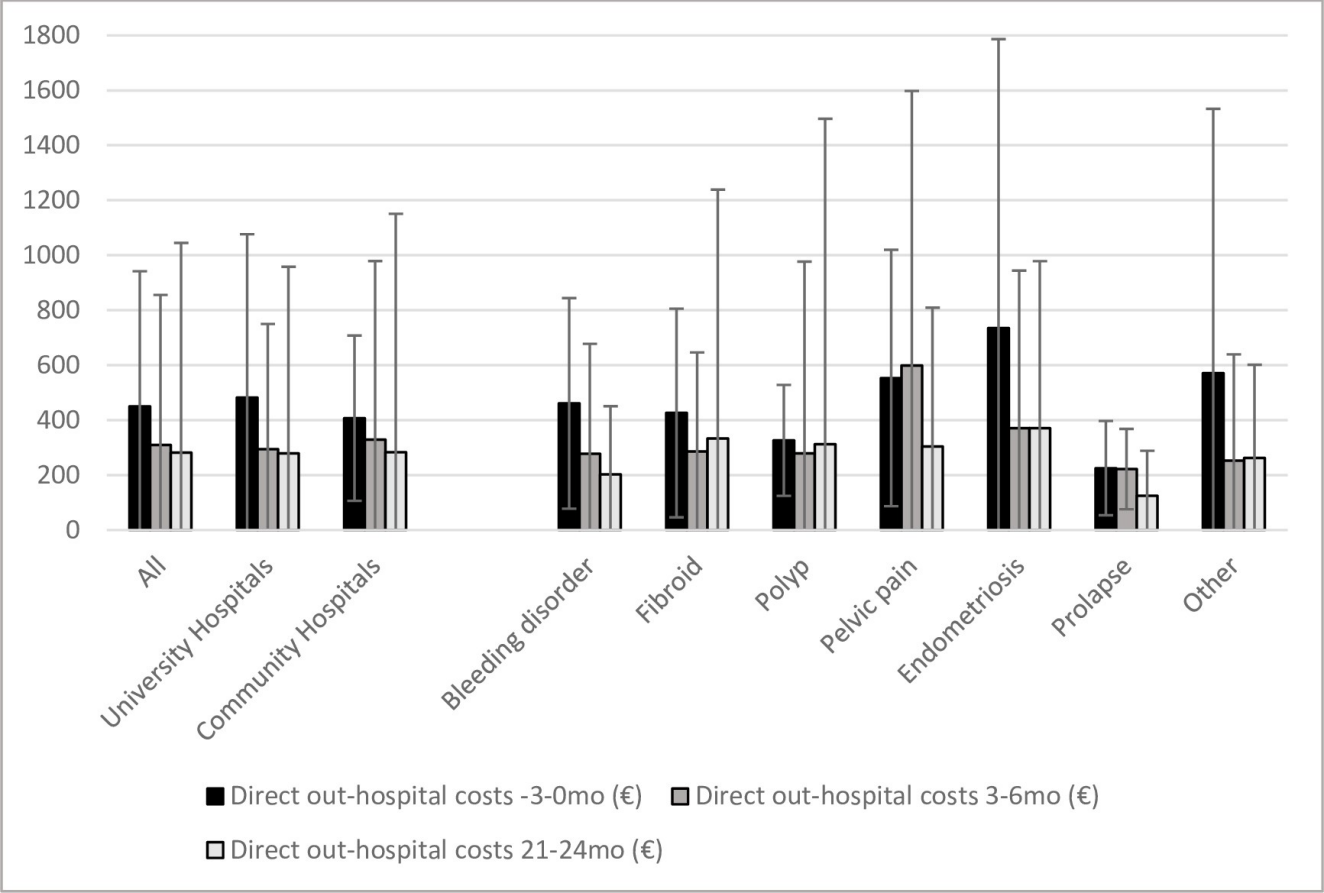
Direct mean out-of-hospital costs (€) according to hospital and diagnosis groups (standard deviation represented by deviation lines).

The highest mean direct costs outside of the hospital were at baseline in the endometriosis group (735.0€ vs. 225.4–570.2€ in the other groups) and at six months in the pelvic pain group (598.3€ vs. 221.8€ - 370.5€). At the two-year follow-up, patients with a bleeding disorder, prolapse or some other benign gynecological condition reported significantly lower direct out-of-hospital costs than at baseline (p<0.05) (Fig 2).

**Fig 3:**
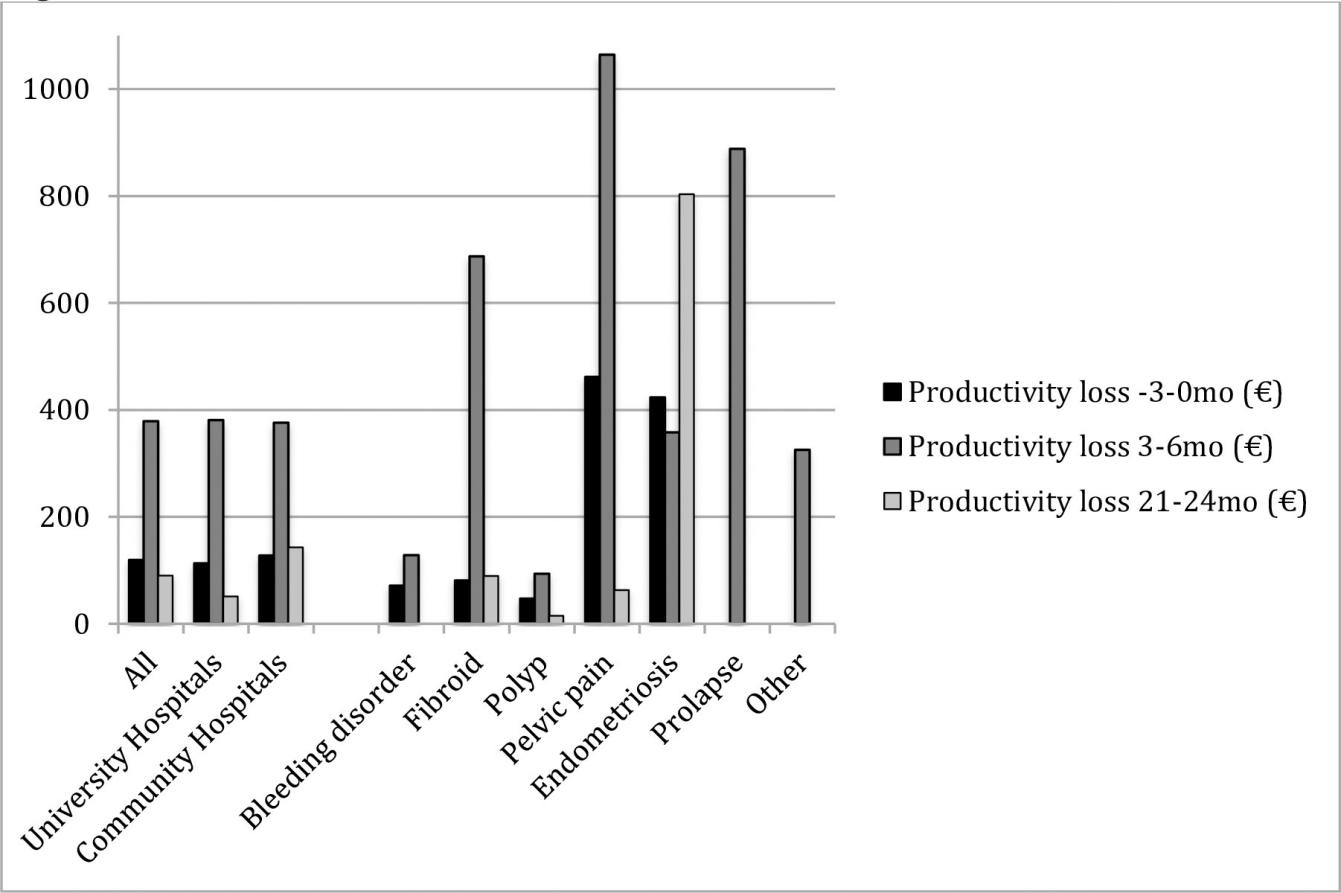
Productivity loss (€) due to sick leave from work according to hospital and diagnosis group.

At baseline, endometriosis and pelvic pain patients (423.7€ and 462.0€, respectively) and at six months patients with fibroids, pelvic pain or prolapse (686.9€, 1064.0€ and 888.0€, respectively) had the highest mean productivity costs compared to the other patient groups. In the long-term, however, marked productivity costs were observed only in endometriosis patients (803.5€ vs. 0–89.3€ of patients in the other groups). A significant decline in these costs during follow-up was noted in patients with a bleeding disorder or pelvic pain (Fig 3).

### 3.5. Distribution of costs outside of gynecological hospitals

Absence from work and ultrasound examinations performed by private gynecologists generated the highest costs outside of the hospital at baseline followed by appointments with general practitioners at primary health care centres or private clinics, LNG-IUD purchases, and appointments with private gynecologists. The burden of sick leave declined from the six-month high back to baseline levels by two years, however, still accounting for the highest proportion of costs outside of the hospital. Already by six months, costs due to private gynecologists’ appointments had, instead, diminished. Appointments with general practitioners at primary health care centres decreased throughout the whole follow-up, whereas costs due to appointments with general practitioners at private clinics and purchases of LNG-IUD persisted throughout the follow-up. Purchases of painkillers, progesterone derivatives, and pharmaceuticals diminishing blood loss decreased from baseline to two years. Cost distribution was otherwise similar in patients of the university hospital and community hospitals throughout the study but only the costs of blood loss diminishing medicines declined significantly in the community hospitals (S2 Fig).

At baseline, patients with pelvic pain used private gynecologists’ services least frequently but visited general practitioners most regularly. At six months, the difference in general practitioners’ visits at primary health care centres prevailed, but there were no longer differences in costs caused by private clinic general practitioner visits or private gynecological services between the diagnosis groups. At two years, also the use of general practitioners’ services at primary health care centres had evened out. At two years, endometriosis patients used private gynecological services significantly more than patients with a bleeding disorder or pelvic pain (S2 Fig).

At baseline, patients with endometriosis or pelvic pain purchased more analgesics than the other diagnosis groups. This tendency persisted throughout the follow-up. At six months, endometriosis patients used significantly more oral progesterone derivatives but fewer bought LNG-IUDs compared to patients with a bleeding disorder. Patients with fibroids used blood loss decreasing medicines significantly more often at the short-term follow-up than patients with polyps. At the long-term follow-up, endometriosis patients purchased more pharmaceuticals to diminish blood loss than patients with a bleeding disorder or a polyp (S2 Fig).

## 4. Discussion

Benign gynecological conditions are often thought to be minor inconveniences rather than actual diseases. However, these conditions greatly affect the quality-of-life of women [1, 8, 9] and medical services are often sought to relieve the discomfort associated with them [7]. Hospital costs, costs to the society, and costs to the patient are significant and similar to those seen in some chronic diseases [10, 11]. As in many chronic diseases, a majority of costs does not arise during the first six months after first hospital contact suggesting a need for continuous healthcare assessment. The costs of treatment of endometriosis seem to be the highest of the gynecological conditions studied [6, 9–12]. Knowledge of the economic burden of the other common benign gynecological conditions is also essential.

Prior studies have shown annual direct hospital costs of gynecological patients to vary from $5400 (€4800, USD costs in 2003 converted to EUR with average USD-EUR exchange rate in 2003) to $9500 (€7640, USD costs in 2005 converted to EUR with average USD-EUR exchange rate in 2005) [4, 10, 11]. The highest annual direct costs reported were those of treatment of endometriosis in the US [$12 118 (€9 110)], and the lowest $1109 (€830) also those of endometriosis, but in Canada [6] (USD costs inflated to 2013 price level using the US Medical Care Consumer Price Index and average USD-EUR exchange rate in 2013). It appears that the average annual costs in our study are lower than those generally reported, and mainly in line with the expenses caused by endometriosis reported from Canada. Due to variable practices, resources, currency, and time frames, cost data from different countries must, however, be interpreted with caution.

Very few studies have analyzed how money is spent in the actual day-to-day environment of gynecological clinics. One previous study with a six-month follow-up from the same area (including only the university hospital) in Finland was published ten years ago [2]. Only patients already scheduled to undergo hysterectomy were included in that study. The direct mean hospital costs were €3114 for endometriosis, €3157 for benign uterine/ovarian cause, €2607 for prolapse and €3553 for menorrhagia. These costs are two to four times higher than those of our operatively treated patients and three to eight times higher than costs of all our study patients (also those treated conservatively) in corresponding patient groups during the six-month follow-up, the biggest difference in costs being in treatment of menorrhagia. Compared to the previously reported results obtained in a selected group of purely operatively treated patients the costs in our patient material, treated with either conservative or operative options, were notably smaller indicating that a mixed approach could be cost saving. The number of hysterectomies has, indeed, decreased in Finland during the early 21^st^ century [17] and bleeding disorders in particular are primarily treated by newer (and cheaper) conservative means. Different surgical techniques, postsurgical treatment protocols and patient characteristics can also contribute to differences in total costs when comparing our study data to the previously reported cost data.

In endometriosis patients, 29% of direct costs have previously been reported to be caused by surgery, 19% by monitoring tests, 18% by hospitalization, and 16% by doctors’ appointments [11]. In our study, almost half of the costs accrued from surgery. Monitoring/diagnostic tests account for a smaller proportion. Hospitalization and doctors’ appointments make up similar proportions as previously reported. As total costs are lower in our study, the absolute amount of money used on surgery per patient is not higher than previously reported. One could speculate that the higher proportion of costs caused by surgery could be a consequence of repeated appointments and monitoring/diagnostic tests having been eliminated from the clinical care process in Finland. It could also be contemplated that hospitalization is more infrequent or postoperative ward treatments shorter, as the same percentage of much smaller total costs is needed to cover these expenses in the gynecological clinics of our study.

Our study showed the treatment of fibroids to be the most expensive in the short-term. By two years, costs of endometriosis and fibroids were the highest. This is in line with two previous studies reporting similar annual costs in these groups [4, 11]. As symptom-causing uterine fibroids are often big (or located submucously), it is understandable that their treatment is expensive due to a high primary surgery rate. The initially low costs of treatment of endometriosis can be explained by the fact that cheaper hormonal treatment is usually the first treatment choice, and only if it fails, one opts for costly operative treatment. As severe cases of endometriosis are often referred to the university hospital, a higher surgery rate and higher costs than in smaller hospitals are not surprising.

Less money, on the other hand, was used on the treatment of polyps in the university hospital. In accordance with previous reports [18, 19], our study also found the treatment of polyps in the operation theatre to be significantly more expensive than ambulatory treatment of them. The difference in costs in the different hospitals is, thus, most likely caused by the higher rate of polyp removal procedures (hysteroscopies) done in the operation theatre in the community hospitals. As surgery increases expenses, it is, out of the economic perspective, reasonable to move procedures that can be done in an outpatient setting away from the operation theatre.

Our study showed that the economic burden outside of the hospital declines after treatment initiation. However, indirect costs due to sick leave do not decrease in the long-term. This finding may indicate a relatively minor need for absence from work in the study group which is reflected in stable productivity costs. The reason why treatment reduces out-of-hospital costs more in patients treated in the university hospital than when treated in community hospitals remains unclear. Since baseline expenses do not differ, the explanation cannot be in different healthcare behaviour in the more rural areas.

Cost data of common benign gynecological conditions in a typical real-world setting, and based on actually realized costs, are rare. Thus, as a strength of the study, our results are valuable for future cost-effectiveness analyses. A small uncertainty is associated with the out-of-hospital costs since these data were gathered by asking patients to recall their use of healthcare services, purchases of pharmaceuticals and days absent from work. In order to minimize bias caused by uncertain memory the surveys were limited to a three-month time period, which also enabled the comparison of equal time periods. Non-medical out-of-pocket costs such as menstrual pads and incontinence pads were unfortunately not gathered from study patients which could also increase the cost burden of some of the study patient groups. The patient material of the study is that of a referral hospital out-patient clinic. The patients were grouped according to clinical assessment using the ICD-10 classification codes based on primary disease. The authors feel that this kind of classification serves clinicians and healthcare assessment better than a symptom based classification as patients most likely present with more than one lead symptom and different diseases present with partly the same symptoms. The authors also feel that this approach may facilitate comparison to data obtained elsewhere as the ICD-10 classification codes are global and often used both in clinical work and scientific studies. Clinical data of women who chose not to participate in the study is unfortunately out of reach of this study. Generally, as clinical practice and resources vary in different countries, cost data should be interpreted by taking into account factors such as local purchasing power parity. As relatively few studies have been published in this field, the possibility of international cost comparison is limited.

## 5. Conclusion

A majority of direct costs does not arise during the first months of treatment but accumulate with time showing the need for prolonged healthcare management and cost follow-up. Lower costs could be achieved by fewer repeated doctors’ appointments, fewer monitoring tests and by re-evaluating the need for hospitalization and length of ward treatment. Procedures that do not need to be done in the operation theatre should be done ambulatorily. Hospital treatment reduces the need to seek medical attention outside of the hospital and decreases out-of-hospital expenses.

## Supporting information

S1 Fig Percentage of patients receiving treatment in the operation theatre according to hospital and diagnosis group. (DOCX)

S2 Fig Cost distribution of out-of-hospital expenses (€) according to hospital and diagnosis group. (DOCX)

S1 Table Research data set. (XLS)
